# Effectiveness of mass vaccination for prevention of hospitalization, severe disease and death due to SARS-CoV-2 omicron Ba.2 variant; A case-population study

**DOI:** 10.1016/j.heliyon.2025.e42670

**Published:** 2025-02-12

**Authors:** Hamid Reza Shamsollahi, Sobhan Younesian, Ali Nikfarjam, Zahra Nasiri, Masud Yunesian

**Affiliations:** a Department of Environmental Health Engineering, School of Public Health, Tehran University of Medical Sciences, Tehran, Iran; b School of Medicine, Tehran University of Medical Sciences, Tehran, Iran; c Deputy of Health, Tehran University of Medical Sciences, Tehran, Iran; d Department of Research Methodology and Data Analysis, Institute for Environmental Research (IER), Tehran University of Medical Sciences, Tehran, Iran

**Keywords:** Vaccine effectiveness, Omicron Ba.2, Case-population study, SARS-CoV-2, COVID-19

## Abstract

One of the primary concerns regarding COVID-19 vaccination programs is the emergence of new virus variants and the effectiveness of the currently available vaccines against these variants. The main objective of this study was to evaluate the effectiveness of the system of vaccination of COVID-19 in Iran in preventing hospitalization, severe illness, critical illness, and death in relation to the Omicron BA.2 variant of SARS-CoV-2. The study focused on assessing vaccine effectiveness regardless of the specific vaccine administered in the community and also investigated the potential improvement in effectiveness after receiving the second dose or subsequent doses of the vaccine. The study specifically examined two age groups including individuals aged 65 years and older and individuals younger than 65 years. This study was performed using case-population method provided by World Health Organization (WHO). To conduct the study, data on vaccination coverage and vaccination status within the community were obtained from the data center of the Public Health Service in the Tehran province, Iran. Additionally, data on hospitalization, critical illness, and death related to COVID-19 were collected from hospitals in Tehran during the period when the Omicron Ba.2 variant was dominant in Iran. The results of the study indicated that vaccination with the available vaccines was effective in preventing severe illness, critical illness, and death resulting from infection with the Omicron variant in both age groups. This study found that completing the vaccination regimen was more effective in preventing adverse outcomes associated with the Omicron variant in elderly individuals compared to younger individuals.

## Highlights

• This study divided cases into two age groups and analyzed comparisons between them.
• Vaccination is generally more effective in younger individuals, while the elderly remain a vulnerable group.
• The increase in VE for the prevention of death, critical illness, and severe illness after completing vaccination is more noticeable in older individuals compared to younger individuals
• Based on these findings, it is evident that increasing vaccination coverage with any available vaccine can be regarded as an effective strategy to reduce the burden of infection caused by the Omicron variant Ba.2 of SARS-CoV-2 in both vulnerable and general populations.

## 1 Introduction

As of April 2024, the coronavirus disease 2019 (COVID-19) pandemic has resulted in over 7 million deaths worldwide. The first confirmed case of COVID-19 in Iran was reported in February 2020. Since then, more than 7.5 million individuals in Iran have contracted the disease, leading to over 150,000 fatalities [1]. To mitigate the spread of infection and reduce mortality rates, various strategies have been implemented since the onset of the pandemic, including social distancing and quarantine measures. However, each of these strategies has exhibited certain limitations and challenges [2].

Mass vaccination against SARS-CoV-2 infection began in early 2021, and since then, concerns have been raised about the effectiveness of these vaccination efforts. The first generation of COVID-19 vaccines were developed based on the Wuhan variant, which was the predominant variant at the time. However, since the start of global vaccination, numerous mutations have been identified in the SARS-CoV-2 genome, particularly in the spike proteins [3,4]. Among these mutations, the Omicron sub-variants have emerged as a significant challenge for mass vaccination. Studies suggest that these variants can evade neutralizing antibodies and exhibit higher transmissibility than the main variant [5].

Another concern regarding mass vaccination is the varied effectiveness of available vaccines, which have been developed using different platforms. The vaccines have shown varying degrees of effectiveness in preventing infection, hospitalization, severe or critical illness, and death [6,7]. The BBIBP-CorV (Sinopharm) vaccine has been the most widely utilized platform in Iran, demonstrating effectiveness rates of 82.5 % against mortality, 65.2 % against hospitalization, and 76.3 % against infection [8]. Additionally, BIV1-CovIran (Barekat), an inactivated whole virus particle vaccine developed domestically, has shown vaccine effectiveness (VE) of 64.9 % against hospitalization and 74.0 % against mortality [9]. The Oxford-AstraZeneca vaccine, another commonly administered vaccine in Iran, exhibited effectiveness rates of 92.0 % against mortality and 65.1 % against hospitalization [8,9].

Moreover, these vaccines’ VE vary against new variants. Additionally, the efficacy of the vaccines are generally lower in elderly individuals compared to younger populations across different vaccine platforms [10]. The use of mixed vaccine regimens with different platforms and structures for the second and third doses in many countries has also raised uncertainties about their effectiveness.

Although evidence suggests a significant reduction in the effectiveness of available vaccines against infection with new variants of COVID-19, the World Health Organization (WHO) maintains that all types of available vaccines can still reduce the risk of severe illness and death caused by new variants, including the Omicron sub-variants [11].

Given these concerns, it is crucial to conduct studies to evaluate the effectiveness of vaccines to ensure their efficacy in mass vaccination campaigns and provide valuable data for vaccine developers, particularly if the development of vaccines targeting new variants becomes necessary.

The Omicron variant and its sub-variants have been most prevalent variant around the world from February 2022 until now [12,13]. In time frame of this study the dominant sub-variant was BA.2. known for its high transmissibility [14]. Based on WHO weekly epidemiological report the total number of confirmed cases was 370,572,213 cases worldwide [15]. Several molecular, epidemiological, and immunological studies have reported milder symptoms associated with the Ba.2 Omicron variant compared to previous variants [[16], [17], [18]]. The incidence of lung involvement is lower than that of previous variants such as Delta, and the severity of signs and symptoms is also reduced [19]. However, concerns still exist regarding hospitalization, severe cases, and death, especially among elderly individuals who are considered a vulnerable group.

Numerous studies have been conducted to evaluate VE against the Ba.2 Omicron variant using various study designs [20]. Many of these studies have focused on the VE of specific vaccine types and many more have assessed the effectiveness in preventing specific outcomes such as death or critical illness. Moreover, age-specific VE has not been consistently reported in these studies [21]. The case-population method is one of the recommended study designs by WHO for determining VE [22]. This approach considers vaccination coverage in general population and the vaccination status of patients, allowing for the estimation of VE for a community regardless of the specific vaccines used or the mixture of vaccines administered. The developed protocol by WHO for the case-population study was followed in this study.

Therefore, the primary focus of this study is to determine the VE against hospitalization, severe illness, critical illness, and death associated with the Ba.2 variant in two age groups: elderly individuals and younger people. Importantly, this assessment was conducted regardless of the vaccine platform, brand, or mixture status of the vaccines administered. By considering all possible combinations of vaccines used, we aim to provide a comprehensive understanding of their effectiveness in preventing these specific outcomes.

## 2 Methods

### 2.1 Study area

The study was conducted in the city of Tehran, Iran. The data used in the study included information on vaccination coverage and recorded cases of hospitalization, severe and critical illness, and death attributed to COVID-19. The data covered the period from February 2022 to April 2022, which coincided with the seventh wave of COVID-19 incidence in Tehran as well as across Iran as a whole. During this time period in both Tehran and Iran, the Omicron Ba.2 variant was the dominant variant of SARS-CoV-2 in Iran.

### 2.2 Study variables and data collection

In this study, the case-population method was employed to assess VE. The primary independent variable in this study is vaccination coverage within the population of the study area, defined as the percentage of eligible individuals who received at least one dose of the COVID-19 vaccine. The dependent variables include hospitalization cases, severe cases, critical cases, and deaths related to COVID-19. Severe cases are characterized by a peripheral oxygen saturation (PO2) level below 90 % and confirmed lung involvement via computed tomography (CT) scans. Critical cases are defined as those requiring intubation due to COVID-19 complications [23].

To obtain data on vaccination coverage, information was derived from the national platform of vaccination data using the national ID code. In Iran, the use of a unique national ID code is mandatory for authentication prior to the administration of vaccinations. This same national ID code is also required for hospital admissions, ensuring a robust system of verification. As a result, this unique national ID code serves as a vital link, seamlessly connecting individual vaccination records with instances of illness and hospitalization. This comprehensive integration significantly enhances the reliability and traceability of our vaccination and healthcare data. The vaccination status of all cases was verified using the national platform of vaccination data. This platform records comprehensive data on the date of vaccination, the number of vaccine doses received, and the type and commercial brand of the vaccine administered for each dose. Specifically, the vaccination coverage data used in the study was obtained from one month prior to the incidence wave of the Ba.2 variant. This allowed for an assessment of the vaccination status of the population at the time when the variant became prevalent.

Additionally, data on individuals who presented to hospitals in Tehran with COVID-like symptoms were collected from the national online platform for recording patients' data in hospitals. This data provided information on hospitalization, severe illness, critical illness, and death related to COVID-19 during the specified time period.

Moreover, data on the most prevalent SARS-CoV-2 variants during the study period were obtained from Iran's SARS-CoV-2 Variant Monitoring Center. This center conducts daily analyses of numerous positive samples, identifying predominant variants through virus sequencing utilizing Next-Generation Sequencing (NGS) technology. The center provided comprehensive data on the dominant variants present during all COVID-19 incidence peaks in Iran.

### 2.3 Data cleaning and sub setting

In the initial step of the study, confirmed cases of COVID-19 were selected from the database. Considering that the minimum age for COVID-19 vaccination in Iran was 12 years, cases under the age of 12 were excluded from the analysis. Subsequently, data on hospitalized cases, severe cases, critical cases, and deaths due to COVID-19 were extracted from the digital database of patients admitted to hospitals in Tehran.

The cases were then categorized into two age groups: individuals younger than 65 years old and those aged 65 years and older (elderly). The vaccination status of all cases was recorded. The cases were further classified into two vaccination categories: those who received only one dose of the COVID-19 vaccine and those who received two or more doses of the vaccine.

VE was calculated using Equation (1):

(1) $$Crude\;VE=1-\left[\left(\frac{PCV}{1-PCV}\right)\left(\frac{1-PPV}{PPV}\right)\right]$$

Where:

VE: Vaccine effectiveness.

PCV: Percentage of cases who are vaccinated.

PPV: Percentage of a comparable group in the population who are vaccinated.

VE was calculated for each group, resulting in a total of four groups based on age and vaccination status.

Uncertainty analysis uses numerical and analytical methods. Analytical methods require known probability distributions and often assume normality, using statistical formulas to propagate uncertainties. Numerical methods, like Monte Carlo, use simulations and sample input parameters from their probability distributions, creating an empirical distribution of the final estimate. This approach provides flexibility for complex systems. Maintaining constant mean and variance in numerical models minimizes the impact of the distribution shape on predictions, ensuring consistent outcomes [24].

Once the point value of VE was estimated for each group, the uncertainty of VE was determined using Monte Carlo analysis assuming a triangular distribution for our input parameters. This analysis enables the calculation of different centiles of VE, and for this study, we specifically selected five points: the minimum value, 5th percentile, median, 95th percentile, and maximum value. These probabilities were reported and presented in a plot, effectively illustrating the range of uncertainty in the VE estimates.

## 3 Results

During the period of this study, the population of the study area, which is Tehran city, was approximately 14,200,000 individuals. Among them, around 13,230,000 individuals were under the age of 65, while approximately 970,000 individuals were classified as elderly (65 years or older). The total number of recorded COVID-19 cases during the study period was 4835 cases.

**Fig. 1:**
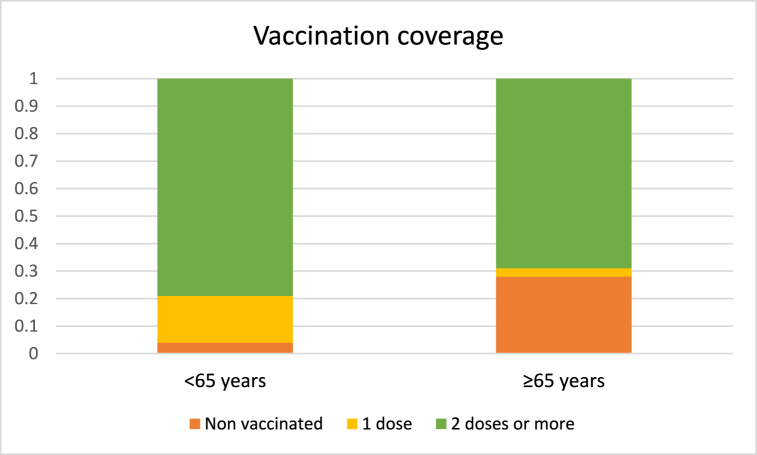
Vaccination coverage percentage in both age groups.

The vaccination coverage status in both age groups is depicted in Fig. 1, which illustrates the distribution of injected vaccine doses. As evident from Fig. 1, despite the prioritization of vaccination for elderly individuals, the percentage of unvaccinated people within the elderly group is approximately seven times higher compared to the younger age group (28 % vs. 4 %). This highlights a significant disparity in vaccination coverage between the two age groups.

According to the Iranian national data sources, until July 2022, a total of more than 206 million doses of vaccines were utilized for mass vaccination. Of these, approximately 162 million doses were imported vaccines, while 45 million doses were domestically produced.

**Fig. 2:**
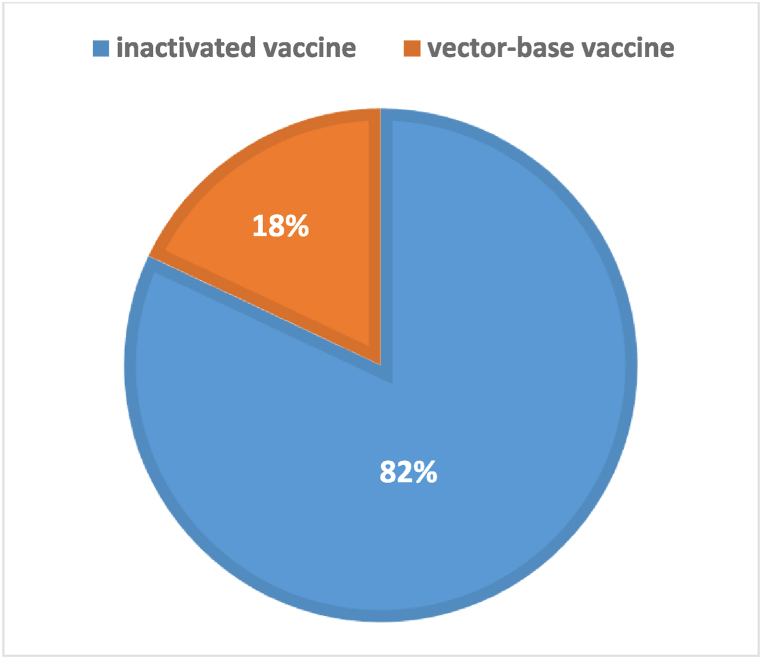
Distribution of vaccine platforms in imported COVID-19 vaccines to Iran (July 2022), Inactivated vaccines: Sinopharm (China) and Bharat (India); Vector-based vaccines: Sputnik (Russia) and AstraZeneca.

The distribution of different vaccine platforms within the imported vaccines can be observed in Fig. 2. It provides information on the relative shares of various vaccine platforms used in the imported vaccines.

**Fig. 3:**
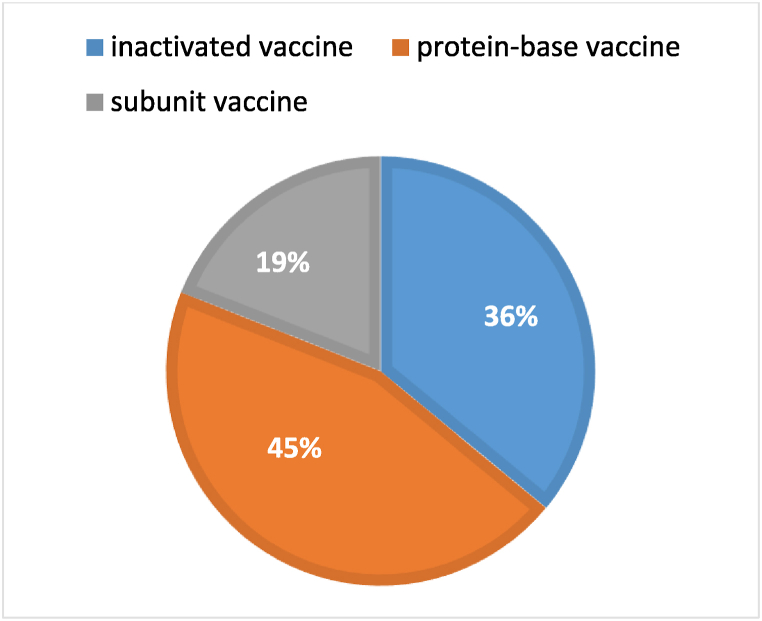
Distribution of vaccine platforms in domestically produced COVID-19 vaccines in Iran (July 2022), Inactivated vaccines: Coviran Barekat and Fakhravac, Protein-based vaccines: Pastocovac, Noora, and Razi, Subunit vaccine: Spikogene.

Similarly, Fig. 3 displays the distribution of different vaccine platforms within the domestically produced vaccines. It illustrates the relative proportions of each vaccine platform used in the domestically produced doses.

**Fig. 4:**
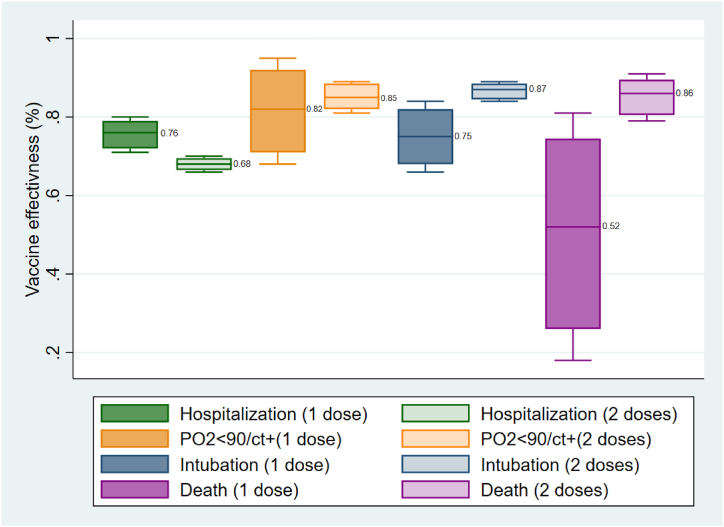
Vaccine effectiveness for prevention of hospitalization, severe illness, critical illness, and death in age group <65years old; PO2: Partial pressure of Oxygen in the blood, CT+: Having confirmed lung engagement via chest CT scan.

**Fig. 5:**
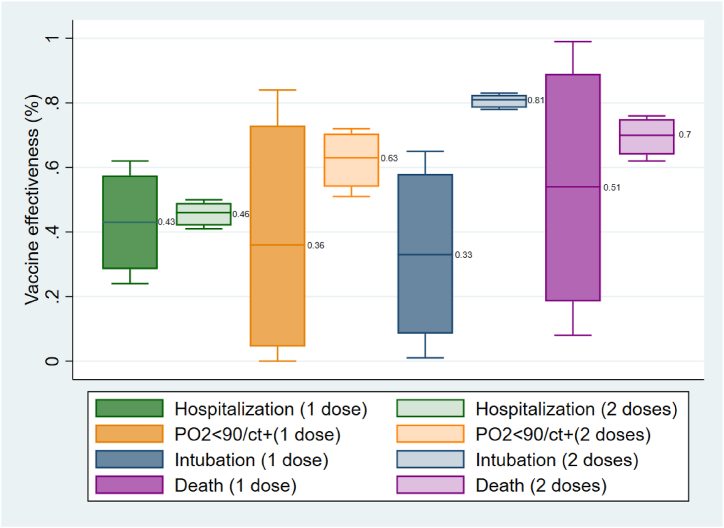
Vaccine effectiveness for prevention of hospitalization, severe illness, critical illness and death in age group ≥65years old.

The estimated VE for the prevention of each outcome (hospitalization, severe disease, critical disease, and death) in both age groups and vaccination status (partially and fully vaccinated), along with their estimated uncertainties obtained through Monte Carlo analysis, are presented in Fig. 4, Fig. 5.

In Fig. 4, the comparison of VE for the prevention of hospitalization in people under 65 years old showed that the VE for partially vaccinated individuals was significantly higher than that for fully vaccinated individuals (P = 0.009) based on the U Mann-Whitney test. However, there was no significant difference in VE for the prevention of severe illness between partially and fully vaccinated individuals (P = 0.834). On the other hand, in this age group, VE was higher in fully vaccinated individuals for the prevention of critical disease (P = 0.012) and death (P = 0.028).

In Fig. 5, for the elderly age group, there were no significant differences in VE for the prevention of hospitalization (P = 0.917), prevention of severe illness (P = 0.602), and death prevention (P = 0.602) between partially and fully vaccinated individuals. However, there was a significant difference in VE for the prevention of critical disease (P = 0.009) between the two vaccination statuses.

## 4 Discussion

This study highlights significant disparities in COVID-19 vaccination coverage and VE between different age groups in Tehran. Despite prioritizing elderly individuals for vaccination, the unvaccinated rate among this group was approximately seven times higher than that of the younger population (28 % vs. 4 %), revealing a considerable gap in vaccination coverage. The results also show varying vaccine effectiveness across age groups and vaccination statuses, with fully vaccinated younger individuals exhibiting higher VE for preventing critical disease and death. However, among the elderly, there were no significant differences in VE for most outcomes between partially and fully vaccinated individuals, except for critical disease prevention. The findings of this study demonstrated the impact of vaccination on reducing death and critical illness.

VE can be measured to assess how well vaccines can prevent death, critical disease, severe disease, hospitalization, or infection caused by SARS-CoV-2 virus. With the emergence of the Omicron Variant, there is growing concern about the level of effectiveness offered by existing vaccines against this new variant. The success of mass vaccination programs, regardless of the specific vaccine used, relies on setting appropriate goals and targets for vaccination.

Numerous studies, including molecular studies, have demonstrated the benefits of vaccination against COVID-19 [25,26]. These studies have indicated that VE for infection prevention may decrease with new variants due to molecular changes in the virus's genome. Despite the reduction in VE for infection prevention across various vaccine platforms, the World Health Organization (WHO) has emphasized that mass vaccination with high coverage remains the primary strategy to protect populations against death and critical illness due to COVID-19 [27]. Therefore, this study focuses on VE for the prevention of death, critical disease, severe disease, and hospitalization specifically related to the Omicron Ba.2 variant regardless of vaccine platform, brand and mixture of doses.

According to Fig. 1, the percentage of non-vaccinated individuals is significantly higher in the elderly group compared to the younger group (P < 0.0001). Despite being at a higher risk of severe COVID-19 infection, more than a quarter of the elderly population remains unvaccinated, even though they were among the first age groups to be prioritized for vaccination. Numerous studies conducted worldwide have examined the willingness to receive the COVID-19 vaccine, and their findings have been controversial. However, many of these studies have reported that the willingness to vaccinate against COVID-19 is lower among the elderly compared to younger individuals. One of the main reasons behind this hesitancy is the concern about vaccine safety and its potential impact on exacerbating symptoms of underlying diseases commonly found in the elderly, such as cardiac diseases, lung diseases, and hypertension. Additionally, worries about the effectiveness of the vaccines have also been cited as a reason for hesitancy [28,29]. It is worth noting that younger individuals with underlying health conditions have exhibited similar behavior [30]. Conversely, the percentage of partially vaccinated individuals, who have received only one dose of the vaccine, is lower among the elderly. This suggests that if the elderly population has trust in the healthcare system and the vaccines, they are more likely to take action and become fully vaccinated.

Death is the most significant outcome of COVID-19 infection. As evident in both Fig. 4, Fig. 5, the VE for death prevention is approximately 50 % in partially vaccinated individuals in both age groups. However, VE for death prevention increases in both age groups among fully vaccinated individuals, reaching over 80 % in individuals under 65 years old and 70 % in individuals aged 65 years and older. These figures demonstrate that the effectiveness of vaccination for death prevention significantly increases following the administration of the second vaccine dose in both age groups, with greater effectiveness observed in younger individuals.

It is worth noting that the vaccination regimen in the study area comprised a mixture of inactivated vaccines, recombinant vaccines, and protein-based vaccines as shown in Fig. 2, Fig. 3. The exact proportions of each vaccine type are unclear, but inactivated vaccines constituted a major part of the administered vaccines in Iran. A matched case-control study conducted in China, which assessed various vaccine platforms and vaccination scenarios against the Omicron variant, reported similar results regarding the increase in VE for death prevention following the second dose of the vaccine. However, the reported VE for death prevention due to the Omicron Ba.2 variant was significantly higher in their study, with VE exceeding 90 % for partially vaccinated individuals and over 95 % for fully vaccinated individuals who received inactivated vaccines across all age groups [31]. Another study focusing on the VE of the mRNA-1273 vaccine reported an effectiveness of 83 % (65–92 %) in preventing ICU admission or death due to Omicron variants across all age groups [32]. None of the mentioned studies specifically assessed VE in different age groups. A study conducted in Hong Kong assessed the VE of BNT162b2 and CoronaVac vaccines across different age groups. The vaccines demonstrated an effectiveness of 71 % (26–91 %) and 91 % (61–98 %) against death in individuals aged 20–59 years, which increased to 84 % (54–96 %) and 93 % (79–98 %) in those aged 60–69 years. These results align with our findings that VE increases with age, although the increase was not statistically significant [33].

The results of this study indicate that vaccination is generally more effective in younger individuals, while the elderly remain a vulnerable group.

Critical illness is considered the second important outcome of COVID-19, defined as confirmed cases requiring mechanical ventilation as a life-sustaining intervention [34]. This definition is consistent with the criteria used in Iran and by the WHO [35]. Fig. 4, Fig. 5 summarize the VE for preventing critical illness in both age groups.

In partially vaccinated individuals, the VE for critical illness prevention is approximately two times higher in the younger age group (<65 years old) compared to the older age group (≥65 years old). Notably, the VE increases to 81 % (90 % uncertainty limit: 78 %–82 %) in fully vaccinated elderly individuals. Other studies focusing on VE against critical illness after mRNA vaccination have reported similar results for fully vaccinated individuals regardless of age groups [36,37]. This is noticeable that the higher observed VE in fully vaccinated younger people comparing elderlies could because of larger population proportion.

Severe illness is also a significant condition among infected individuals. It is defined as a patient requiring oxygen therapy due to a drop in PO_2_ levels below 90 %, confirmed by chest CT scan showing lung involvement [38]. According to our results, the VE in preventing severe illness was more than two times higher in the younger age group compared to the older age group in partially vaccinated individuals (82 % vs. 36 %). Completion of vaccination did not significantly increase VE in the younger age group (mean VE increased from 82 % to 85 %). However, in the older age group, VE increased from 36 % to 63 % after full vaccination. Among fully vaccinated individuals, the mean VE in the older age group was still lower than in the younger age group. Nevertheless, full vaccination had a significant effect in reducing the risk of severe illness in the older age group.

The impact of vaccination completion on the prevention of hospitalization showed the weakest observed effect. Among partially vaccinated individuals, VE for preventing hospitalization was 76 % in younger individuals and 43 % in older individuals. Surprisingly, VE decreased to 68 % in younger individuals after full vaccination. Additionally, the increase in VE for older individuals was not prominent, going from 43 % to 46 % among fully vaccinated individuals (P value: 0.917). This phenomenon could be attributed to the prevalence of COVID-19 in the population. According to the WHO, in order to efficiently screen for SARS-CoV-2 infected individuals, the percentage of positive COVID-19 tests should not exceed 3 % of daily tests [39]. However, data from the WHO COVID-19 dashboard suggests that in February 2022, this percentage was nearly 30 % in Iran. As a result, a significant number of symptomatic cases went undetected by confirmed tests. Additionally, since many COVID-19 cases are asymptomatic, there is no need for these individuals to undergo testing. So, it is expected that many individuals who received one dose of the vaccine had been infected before vaccination. This means they had already been exposed to the antigen through infection and subsequently received one dose of the vaccine, resulting in a stronger immune response compared to individuals who had only one exposure to the antigen through vaccination. This could potentially explain why the VE for hospitalization prevention was not significantly improved in fully vaccinated individuals, particularly in younger individuals.

The reported VE for the prevention of hospitalization due to the Omicron variant in other studies generally exceeds the findings of this study. For instance, a study conducted in South Africa reported a VE of 61 % for hospitalization prevention one month after individuals received two doses of the BNT162b2 mRNA vaccine [40]. It is important to note that while most studies report VE for hospitalization prevention across various age groups with a narrower age range, this study divides cases into two age groups due to the small number of cases. Consequently, the available data on VE is limited only to these two age groups. Furthermore, the presence and history of underlying diseases in COVID-19 cases may vary across different countries. Therefore, comparing the results of this study with other studies becomes challenging and complex.

Maximizing vaccination coverage and ensuring full vaccination of vulnerable individuals is a key strategy to achieve vaccination goals and prevent severe illness, as emphasized by the WHO in its policy brief on vaccination targets [41]. In a systematic review on intubated COVID-19 patients (critical illness), it was found that 70 % of these patients required cardiopulmonary resuscitation (CPR). The fatality rate in this group was 97.5 %, with only 2.5 % surviving [42]. Therefore, the prevention of critical or severe illness becomes a crucial objective of vaccination.

According to the findings of this study, vaccination with any available vaccine maybe effective in preventing critical and severe illness and death. The effectiveness further increases after the second dose of the vaccine and is generally higher in younger individuals. Notably, the improvement in effectiveness percentage after the second dose for preventing critical illness is significant in older individuals aged 65 and above. Based on these findings, it is evident that increasing vaccination coverage with any available vaccine can be regarded as an effective strategy to reduce the burden of infection caused by the Omicron variant Ba.2 of SARS-CoV-2 in both vulnerable and general populations [43].

### 4.1 Limitations

This study had several limitations that need to be acknowledged. Firstly, the vaccination coverage for each gender was not available, which prevented the determination of VE for both genders. Additionally, the specific details regarding the mix and match regimen for booster doses in individuals were unclear, making it impossible to assess VE for different combinations of vaccines. Due to significant challenges in obtaining COVID-19 vaccines, there were numerous variations in vaccine combinations regarding platform, dose number, sequencing, timing, and commercial brand. These mix-and-match scenarios were not predetermined but resulted from vaccine availability and individual preferences. Consequently, assessing the effectiveness of each vaccine platform was not feasible. However, this was not the aim of our study. Our objective was to evaluate the general vaccination program in preventing hospitalization and mortality. Furthermore, it is important to note that this study specifically focused on the Ba.2 variant of SARS-CoV-2. The VE observed in this study may differ when considering other variants of the virus.

## 5 Conclusion

Indeed, increasing vaccination coverage, regardless of the vaccine platform, is an effective strategy for preventing death, critical illness, and severe illness caused by the Omicron variant of SARS-CoV-2. The VE for the prevention of death, critical illness, and severe illness is generally higher in the age group below 65 years compared to older individuals. However, the increase in VE for all these outcomes after completing vaccination (receiving the full vaccination with the second dose or more) is more noticeable in older individuals compared to younger individuals. Therefore, full vaccination is particularly crucial for protecting older individuals from severe COVID-19 outcomes.

## CRediT authorship contribution statement

**Hamid Reza Shamsollahi:** Writing – original draft, Project administration, Methodology, Funding acquisition. **Sobhan Younesian:** Writing – review & editing, Formal analysis. **Ali Nikfarjam:** Data curation. **Zahra Nasiri:** Visualization, Formal analysis. **Masud Yunesian:** Supervision, Methodology, Investigation, Formal analysis, Conceptualization.

## Data sharing

All of the finding data is shared in this article.

## Ethical declarations

Since we utilized secondary data that had been deidentified and was not linked to any individual identities, obtaining informed consent was neither feasible nor required. The confidentiality of the participants was adequately protected. Additionally, measures were implemented to ensure that the data were not publicly disseminated and were not freely accessible. The ethical approval of this study is available in Iran national committee for ethics in biomedical research by ethical codes of IR.TUMS.MEDICINE.REC.1401.058.

## Funding

Tehran University of Medical Sciences, Tehran, Iran. The grant number is 1401-1-129-57435.

## Declaration of competing interest

We want to express that this manuscript has not been previously published in other journals. All the authors have contributed to the development of the manuscript and the analysis behind it. We have no conflict of interests. Please do not hesitate to contact us if any further details are required.
